# A rare presentation of an acute abdomen: an ileal diverticular perforation

**DOI:** 10.1186/s13104-017-2514-z

**Published:** 2017-06-02

**Authors:** Basuru Uvindu Thilakawardana, Sanjay De Mel, Vasitha Abeysuriya, Janaki Hewavisenthi, Chandima De Mel, Lal Chandrasena, Visula Abeysuriya

**Affiliations:** 1 Nawaloka Hospitals Research and Educational Centre, Nawaloka Hospitals PLC, No. 23, Deshamanya H.K. Dharmadasa Mw, Colombo 02, Sri Lanka; 2 Faculty of Medicine Ragama, University of Kelaniya, Kelaniya, Sri Lanka

**Keywords:** Ileal, Perforation, Laparoscopy, Diverticular

## Abstract

**Background:**

This case report highlights the value of prompt intervention of diagnostic laparoscopy in a patient suspects of having an acute abdomen due to an intestinal perforation, where there is a limitation of performing Contrast Enhanced Computed Tomography of abdomen. A previously healthy young adult presenting with an acute abdomen due to a spontaneous ileal perforation, without any associated risk factors is a rare clinical entity in a developing country. Therefore, entertaining an early diagnosis will possibly prevent a fatal consequence.

**Case presentation:**

A male patient, 29 years old, recently diagnosed as a young hypertensive without any associated factors, currently on antihypertensive treatment, was admitted to our hospital presenting with an acute severe abdominal pain. During initial assessment, the patient was febrile (101 °F), ill looking, tachycardic (pulse rate 121 bpm) with rapid shallow breathing. Abdominal examination reviled diffuse guarding and rigidity, more severe on right iliac fossa. Following history and clinical examination probable clinical diagnosis was made as an acute appendicitis with perforation. However, ultrasonography was found to have normal appendix. Contrast Enhanced Computed Tomography was not performed as a subsequent investigation because of the impairment of renal functions of this patient. Though, non-contrast CT would have been ascertained more diagnostic yield, given the critically ill status of this patient we decided to perform urgent diagnostic laparoscopy. It reviled pus in several abdominal cavities and dense adhesions. Therefore, the procedure was converted to a laparotomy and found to have an ileal perforation with diffuse peritoneal contamination. Diseased ileal segment was resected and anastomosed. Followed by peritoneal lavage.

**Conclusion:**

Ileal perforation due to diverticular disease in a healthy young adult is rare. This case report highlights the importance of considering this clinical entity as a differential diagnosis, the value of early diagnostic laparoscopy, especially in clinical settings with limitations to CT scan, since late diagnosis can give rise to fatal outcome.

## Background

The causes of Ileal perforation are many. According to literature the commonest cause is spontaneous ileal perforation [1]. Other causes are tuberculosis, typhoid, lymphoma, immunocompromisation, trauma, long standing steroid therapy and bowel obstruction. The incidence of ileal diverticular perforation is rare [1, 2]. Diagnosis of ileal perforation can be made accurately by Contrast Enhanced Computed Tomography (CECT) scan of the abdomen and pelvis as it demonstrates the site of perforation [3]. However, clinical diagnosis of acute abdomen due to ileal perforation in a young male adult, with non-progressive renal disease with increased level of serum creatinine is challenging. Therefore, it is good practice to consider diagnostic laparoscopy at an earlier stage [4]. The Ileal and Jejunal diverticular disease is rare than diverticuli in duodenum and colon. Even the most published literature on jejunum and ileal diverticular disease stated of pseudodiverticuli than true diverticuli [4, 5]. The occurrence of complicated diverticular disease is common among the elderly [6]. To our knowledge, the presentation of a young adult with an acute abdomen due to a true ileal diverticular perforation with difficulty in clinical diagnosis due to restrictions of CECT abdomen due to hypercreatininemia with non-progressive renal disease is a clinical challenge. Failure of early diagnosis of perforated ileal diverticuli can be fatal [6].

## Case report

Twenty nine year old male patient with persistent elevated blood pressure who was 2 weeks ago newly diagnosed of having non progressive renal disease with elevated creatinine levels. The patient presented to the surgical ward complaining of right sided lower abdominal pain with two to three episodes during last 5 days after his initial medical diagnosis.

He has been recently well until his renal disease was diagnosed. No significant surgical history and no medical co-morbidities other than renal disease, no significant family history of renal and gastrointestinal disease, no immunocompromised state no recent trauma. He was put on low dose steroids for 2 weeks. And his hematological, radiological and biochemical investigations had not shown any features of malignancy during his initial diagnosis of renal disease 2 weeks ago.

On clinical examination, the patient was febrile (101 °F), ill looking, tachycardic (pulse rate 121 bpm) with rapid shallow breathing. Abdominal examination reviled diffuse guarding and rigidity, more severe on right iliac fossa. It was found he was having acute abdomen with sepsis. Subsequent hematological and biochemical investigations showed inflammatory process. Initial full blood count reviled, white blood cell count of 23,050/mm^3^, with 94.6% of Neutrophils, 2.9% of Lymphocytes, 0.0% of Eosinophils, 2.5% of Monocytes, 0.0% of Basophils. His Initial hemoglobin was 11 g/dl and Platelet count was 226,000/mm^3^. The provisional working clinical diagnosis was suspected of him having a perforated appendix. However, ultrasound scan of the abdomen did not show any evidence of inflamed appendix since the ultrasonic outer diameter of the appendix was not exceeding more than 6 mm. Except pelvic, right iliac fossa, right para colic gutter fluid collection. The multidisciplinary discussion concluded that the patient need to undergo a diagnostic laparoscopy since the CECT is restricted due to his non-progressive renal disease and hypercreatininemia. Diagnostic laparoscopy was performed with sub umbilical approach, with Open Hassan Technique with 10 mm trocha with 2.5 l of Carbon Dioxide insufflation with 12 mmHg of intra-abdominal pressure. 10 mm, 30 degree side view scope was used. During laparoscopy, diffused adhesions with intestinal content and pus was noted in the pelvis, right iliac fossa, left iliac fossa, and right paracolic gutter. Appendix macroscopically found not inflamed. Rest of the laparoscopic peritoneal survey found normal. Due to difficult anatomical visualization laparoscopy was converted to a lower midline laparotomy and small bowel survey was done, ileal perforation was noted 25 cm proximal to the ilio-colic valve. Rest of the peritoneal survey was found normal. No diverticular disease was found in rest of the gastro intestinal tract. Thorough peritoneal washing was done with copious amount of 0.9% warm saline. Ileum was resected, distal and proximally 2 cm away from the perforated site and side to side anastomosis was established by using 60GIA Boston Scientific Stapler (Echelon Flex™ Endopath^®^ staplers, Ethicon US, LLC, 2010/2016) with a pelvic drain in situ. Patient was in intensive care unit with supportive therapy for 3 days and had an uneventful recovery. And discharged on fifth post-operative day and presently in the surgical clinic follow up. Surgically resected specimen was sent for histological analysis. Specimen was a piece of bowel measuring 15 mm × 10 mm × 5 mm. The entire specimen has been sampled. Microscopy reviled sections of small intestine displaying a normal crypt villous architecture with no evidence of villous flattening. There was no evidence of significant inflammation within the lamina propria. The mucosa-muscularispropria contains a diverticulum surrounded by extensive acute and chronic inflammation leading to the serosa of the bowel. Hence denoting the presence of diverticulum inflammation and perforation. There is no evidence of a neoplasm or presence of inflammatory bowel disease.

## Discussion

Literature projects the prevalence of small intestinal diverticular disease is 0.06–1.3% [7]. This case report is a good example to emphasize that there can be rare causes for acute abdomen such as spontaneous intestinal perforation [1]. This patient has no history of trauma and as we mentioned above he presented with right iliac fossa pain and tenderness which mimics acute appendicitis. As he was recently diagnosed with a renal disease and was on low dose corticosteroids, it was a concern whether this pain is due to a renal pathology also. It has been proven that some drugs can cause small intestinal ulceration and rarely perforation [8, 9]. Among them, corticosteroids responsible for intestinal perforation [10]. As ultrasonography excluded appendicular pathology, and it is clinically suggestive of intestinal perforation, next investigation of choice was a CECT scan of abdomen and pelvis [1]. His serum creatinine was high (3.3 mg/dl) due to an undiagnosed reason which made us to perform a diagnostic laparoscopy which reviled pus in abdominal cavity. Therefore, the clinical suspicion of intestinal perforation was made. Primary cause which led to intestinal perforation was a concern. Typhoid fever is a known cause for intestinal perforation [11]. Incidence of small bowel diverticuli are twice frequent in men than women. It is more common in elderly and it has been found that distally occurring diverticuli are smaller and fewer in number and vice versa in small intestine. Percentage of incidence can be projected as 75% in proximal jejunum, 20% in distal jejunum and 5% in the ileum [12]. It is important to note that although diverticular disease is more common in elderly it also can occur in young age and can have a fatal presentation. In this case, intraoperatively there was no suspicion of diverticular disease as there was no diverticuli found in small and large intestine. That points towards the conclusion that the perforated diverticulum is the only diverticulum he had in his intestine and it got perforated which is an extremely rare incident.

## Conclusion

We would like to suggest performing diagnostic laparoscopy for suspected intestinal perforation where CECT is not available may results in early diagnosis of intestinal perforation, hence avoid fatality. And also, it is worth to notice that thorough peritoneal washing opens doors to patients with pus and intestinal content in peritoneum to have side to side intestinal anastomosis with no postoperative complications.

## Abbreviation

CECT: Contrast Enhanced Computed Tomography
